# Improving Medical Imaging with Medical Variation Diffusion Model: An Analysis and Evaluation

**DOI:** 10.3390/jimaging9090171

**Published:** 2023-08-25

**Authors:** Zakaria Rguibi, Abdelmajid Hajami, Dya Zitouni, Amine Elqaraoui, Reda Zourane, Zayd Bouajaj

**Affiliations:** Research Laboratory Watch Laboratory for Emerging Technologies (LAVETE), Hassan First University of Settat, Settat 21000, Morocco; abdelmajid.hajami@uhp.ac.ma (A.H.); zitouni.dya@uhp.ac.ma (D.Z.); a.elqaraoui@uhp.ac.ma (A.E.); zouranereda333@gmail.com (R.Z.); zayd.bouajaj@esi.ac.ma (Z.B.)

**Keywords:** variational diffusion models, medical imaging

## Abstract

The Medical VDM is an approach for generating medical images that employs variational diffusion models (VDMs) to smooth images while preserving essential features, including edges. The primary goal of the Medical VDM is to enhance the accuracy and reliability of medical image generation. In this paper, we present a comprehensive description of the Medical VDM approach and its mathematical foundation, as well as experimental findings that showcase its efficacy in generating high-quality medical images that accurately reflect the underlying anatomy and physiology. Our results reveal that the Medical VDM surpasses current VDM methods in terms of generating faithful medical images, with a reconstruction loss of 0.869, a diffusion loss of 0.0008, and a latent loss of $5.740068\times 10^{-5}$. Furthermore, we delve into the potential applications of the Medical VDM in clinical settings, such as its utility in medical education and training and its potential to aid clinicians in diagnosis and treatment planning. Additionally, we address the ethical concerns surrounding the use of generated medical images and propose a set of guidelines for their ethical use. By amalgamating the power of VDMs with clinical expertise, our approach constitutes a significant advancement in the field of medical imaging, poised to enhance medical education, research, and clinical practice, ultimately leading to improved patient outcomes.

## 1. Introduction

Medical imaging is a critical tool in modern medicine, enabling clinicians to diagnose and monitor a wide range of conditions. However, image analysis can be challenging due to noise, artifacts, and other sources of variability. To address these challenges, variational diffusion models (VDMs) have been developed as powerful tools for image processing and analysis. VDMs are based on partial differential equations that allow for the smoothing of images while preserving edges and other important features [1,2].

In this paper, we propose a novel approach to medical imaging based on VDMs, which we refer to as the Medical VDM. The objective of the Medical VDM is to enhance the accuracy and dependability of medical image analysis. Our approach is uniquely designed to take full advantage of the specific imaging modality being used, thereby offering more robust and reliable analysis.

We begin by reviewing the relevant literature on VDMs and medical imaging, including previous research on the use of VDMs in medical applications [3,4]. We then describe the Medical VDM approach in detail, including its mathematical basis and the advantages it offers over existing VDM approaches. Next, we present experimental results demonstrating the effectiveness of the Medical VDM for medical image analysis, comparing its performance with other state-of-the-art methods. We also discuss the potential applications of Medical VDM in clinical practice and highlight areas for future research.

So far, we have explored three categories of generative models: GAN, VAE, and flow-based models. Although they have demonstrated success in producing high-quality samples, each model comes with certain drawbacks. GAN models can suffer from instability during training and might produce less diverse outputs due to their adversarial approach. VAEs depend on an alternative loss function, while flow models necessitate unique structures for achieving reversible transformations.

Diffusion models draw inspiration from non-equilibrium thermodynamics and employ a Markov chain consisting of diffusion steps to incrementally introduce random noise into data. These models then learn to undo the diffusion process in order to generate desired data samples from the noise. Distinct from VAE and flow models, diffusion models feature a set learning process and a high-dimensional latent variable. There have been several proposed diffusion-based generative models, including denoising diffusion probabilistic models [5,6].

Recent years have witnessed a growing interest in applying variational diffusion models to medical imaging, as evidenced by a number of related works published in top-tier conferences and journals. For instance, Kazerouni et al. [7] proposed a survey with the intention to provide a comprehensive overview of diffusion models in the discipline of medical image analysis. Similarly, Wolleb et al. in the paper [8] intended to provide a good application of diffusion models in the discipline of medical image for anomaly detection. Another notable example is the work by Xia et al. [9], who introduced a conditional denoising diffusion probabilistic model (DDPM) to improve the low-dose computed tomography denoising performance, demonstrating encouraging results with a high computational efficiency. These studies highlight the potential of VDMs in enhancing the accuracy and efficiency of medical imaging applications and pave the way for further exploration of this promising research direction.

In the referenced paper [10], the authors investigate the generation of synthetic images derived from high-resolution 3D brain images using latent diffusion models. They trained their models on T1w MRI images from the UK Biobank dataset, which includes 31,740 images, to understand the probabilistic distribution of brain images based on covariables, like age, sex, and brain structure volumes. The results revealed that their models were capable of generating realistic data, with the ability to effectively manipulate data generation using conditioning variables. Moreover, they developed a synthetic dataset comprising 100,000 brain images and made it accessible to researchers.

Over the past few years, there has been a surge in publications exploring the use of variational diffusion models in medical applications. Notable works include those by Sohl-Dickstein et al. [11], Chen et al. [12], Ho et al. [5], Rezende and Mohamed [13], among others [14,15,16,17].

The remaining sections of this paper are structured as follows: In Section 1, we present an introduction to the Medical VDM and its relevance in the field of medical image generation. In Section 2, we discuss the methods and the process of accurate and reliable medical image generation using variational diffusion models. Section 3 is dedicated to presenting the results and the analysis of our proposed approach we also delve into the discussion and potential future directions for this research. Lastly, we conclude our findings and summarize the key points of this study in Section 4.

The term Medical VDM refers to medical image generation using variational diffusion models. We will often use the abbreviation VDM when discussing this particular approach in this paper.

## 2. Methods

In this work, we aim to apply the VDM model to generate medical images. We employed a simulation-based approach to assessing the performance of the Medical VDM in generating high-quality medical images. Our dataset comprised 1341 medical images sourced from the National Institutes of Health Chest X-ray Dataset [18]. This dataset contains over 100,000 chest X-ray images, annotated with various pathologies. It covers a wide range of patient demographics, ensuring the generalizability of our results.

The images were preprocessed by resizing them to a standard resolution of 256 × 256 pixels, followed by normalization to the range [0, 1], without any data augmentation techniques. We implemented a custom Medical VDM architecture, which leverages a variational diffusion model to smooth images while preserving important features, such as edges. The forward and reverse processes were designed to transform medical images into Gaussian noise and reconstruct them, respectively.

In our implementation, we employ the AdamW optimizer with a combination of a linear warmup and cosine decay schedules for more effective and stable learning. We first apply a cosine decay schedule with an initial learning rate of 1.0, a total number of steps TSTEPS, and a final learning rate of $1\times 10^{-5}$. Next, we use the AdamW optimizer with a learning rate of $8\times 10^{-4}$, first and second moment coefficients (b1 and b2) set to 0.9 and 0.99, respectively, an epsilon value of $1\times 10^{-8}$, and a weight decay of $1\times 10^{-4}$. Finally, we apply a linear schedule that increases the learning rate from 0.0 to 1.0 over 250 steps. We then initialize our parameter storage with the given parameters and optimizer state, a random number generator (RNG), and an iteration counter set to 0. The storage is replicated across devices using the replicate function for distributed training. A batch size of 1t was used and we trained the model for T to equal 20 K time steps globally.

The Medical Variational Diffusion Model (M-VDM) architecture consists of an encoder with 6 ResNet blocks for both content and time embedding, a decoder with 6 ResNet blocks, and a score network. The model operates with a latent space dimension of 64. It employs a gamma range of 0.1 to 1.0 with 50 linearly spaced steps for the diffusion process, where each step is divided into 1000 substeps. The total number of time steps is set to 5. The model’s hyperparameters also include a learning rate of 1 × 10^−4^, a batch size of 64, and a weighting factor for the losses. These values have been empirically determined to ensure effective training and the generation of high-quality samples. It is important to note that these hyperparameters can be further tuned based on the specific dataset and task requirements to achieve optimal results.

The loss function used for training was a combination of mean squared error (MSE), Kullback–Leibler divergence (KL divergence), which measures the difference between the predicted distribution and the target distribution, and differential loss, which measures the gradient difference between the original and the reconstructed image. By minimizing the differential loss during training, the model learns to generate images with smooth transitions and avoid sharp edges. This loss is commonly used in image generation tasks, where the generated image must have smooth transitions and should not have sharp edges.

The VDM class is designed as a neural network module and consists of several components and methods to perform various tasks, such as reconstruction loss, latent loss, and diffusion loss computation. The class also includes methods for encoding, decoding, and embedding, which are essential for transforming and processing medical images.

The VDM class has several adjustable parameters, such as the number of time steps, gamma min, gamma max, embedding dim, layers, and classes. These parameters can be fine-tuned for an optimal performance in generating medical images. The class also offers antithetic time sampling as an option to improve the efficiency of the process.

The VDM class is implemented using JAX, a high-performance machine learning library that combines Autograd and XLA for efficient research purposes. This approach allows for transparency and reproducibility, enabling the scientific community to build upon our findings and further advance the field of medical imaging.

Turning our focus to the fundamental principles of generative modeling, the process involves estimating the marginal distribution p(x) using a dataset comprising observations of x. The methods described can be extended to multiple observed variables and/or the task of estimating conditional densities p(x|y), which is common in generative models. Our proposed model includes a diffusion process (Section 2.1) that we invert to obtain a hierarchical generative model (Section 2.3). Remarkably, we show that the model choices lead to a simple variational lower bound (VLB) of the marginal likelihood, which we use to optimize the model parameters.

### 2.1. Forward-Time Diffusion Process

In this section, we introduce a forward-time diffusion process that serves as a building block for our proposed latent-variable model. This process involves iteratively applying a diffusion operator to a given initial density function, which results in a sequence of density functions. We show that, under certain assumptions, this diffusion process can be inverted to obtain a generative model that maps from a latent space to the observed data space. Specifically, we derive a closed-form expression for the generative model that involves integrating the diffusion operator along a reverse-time trajectory.

**Figure 1:**
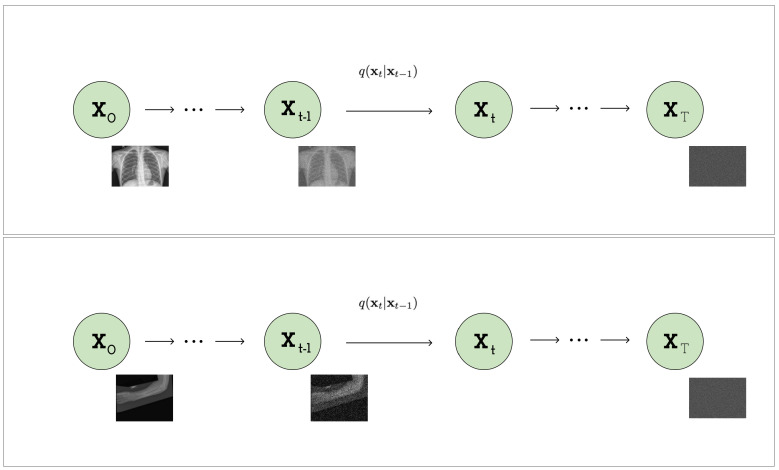
Visual representation of the forward VDM process: constructing a noisy Image from a medical image via noise addition over time.

To apply the VDM model to medical imaging (see Figure 1), we start with a Gaussian diffusion process that begins with the observed medical images (*x*) and defines a sequence of increasingly noisy versions of *x*, called *latent variables ($z_{t}$)*, where *t* ranges from 0 (least noisy) to 1 (most noisy). The distribution of latent variable $z_{t}$ conditioned on *x* for any t∈[0,1] is determined by a scalar-valued function of *t* ($\alpha_{t}$) and a strictly positive scalar-valued function of *t* ($\sigma_{t}^{2}$), which are both assumed to be smooth with finite derivatives with respect to *t*.

We define the signal-to-noise ratio (SNR) as $\alpha_{t}^{2}/\sigma_{t}^{2}$, where the SNR is strictly monotonically decreasing in time to reflect the increasing noise of the latent variables as we move forward in time.

We use the variance-preserving diffusion process as a special case, where $\alpha_{t}=\sqrt{1-\sigma_{t}^{2}}$, and the distributions $q(z_{t}|z_{s})$ for any t>s are also Gaussian. The joint distribution of latent variables at any subsequent time steps is Markov. The distributions $q(z_{s}|z_{t},x)$, for any 0≤s<t≤1, can be calculated using the Bayes rule and are also Gaussian.

### 2.2. Noise Schedule

**Figure 2:**
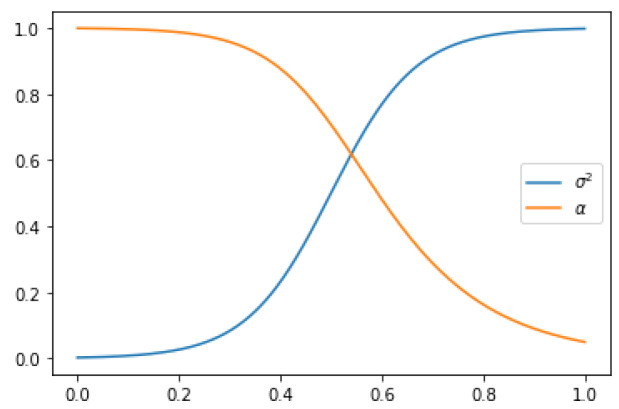
Visual representation of the forward VDM process: transforming a medical image into Gaussian noise through incremental noise addition over time.

In this study, we present an approach (inspired by [5], as depicted in Figure 2) to the noise schedule, which involves learning the annealing parameters from data. Specifically, we use a monotonic neural network to parameterize the variance of the diffusion process as a function of time, such that the noise level of the latent variables gradually increases over time.

This approach allows the model to adapt to the complexity of the data and capture increasingly fine-grained features as the noise level increases. In our experiments, we use a variance-preserving diffusion process, where the signal-to-noise ratio is a decreasing function of time. We show that the learned annealing parameters result in a simple form for the signal-to-noise ratio, which can be used to optimize the model parameters efficiently.

### 2.3. Reverse-Time Generative Model

The generative model consists of a sequence of distributions defined at each time step in reverse order, from the noisiest observation to the least noisy one, where the variable “Xt” represents the observation at time step “t” in the generative model:

$$\begin{array}{cc} p_{\theta }\left(x_{T}\right) & =N(x_{T}|0,I)\\ p_{\theta }\left(x_{t-1}|x_{t}\right) & =N(x_{t-1}|f_{\theta }\left(x_{t},\epsilon_{t-1}\right),\sigma_{t-1}^{2}I)\\ p_{\theta }\left(x_{t-2}|x_{t-1},x_{t}\right) & =N(x_{t-2}|f_{\theta }\left(x_{t-1},\epsilon_{t-2}\right),\sigma_{t-2}^{2}I)\\ \; & \vdots \\ p_{\theta }\left(x_{0}|x_{1},\ldots ,x_{T}\right) & =N(x_{0}|f_{\theta }\left(x_{1},\epsilon_{0}\right),\sigma_{0}^{2}I) \end{array}$$

where $\epsilon_{t}$ is a noise vector sampled from a fixed distribution, $\sigma_{t}$ is a scalar parameter that controls the variance of the distribution at each time step, and $f_{\theta }$ is a neural network that maps its inputs to the mean of the distribution. The joint distribution of the latent variables and observations is then given by the following:

(1) $$p_{\theta }\left(x_{0:T},z_{1:T}\right)=p_{\theta }\left(x_{0}|z_{1}\right)\prod_{t=1}^{T}p_{\theta }\left(x_{t}|x_{t-1},z_{t+1:T}\right)p_{\theta }\left(z_{t}|x_{1:t}\right)$$

where $z_{t}$ is the latent variable at time step *t*.

The distribution $p_{\theta }\left(z_{t}|x_{1:t}\right)$ is obtained by running the diffusion process in reverse, using the same noise schedule and the same neural $p_{\theta }$ but in reverse order:

$$\begin{array}{cc} z_{T} & =x_{T}\\ z_{t-1} & =f_{\theta }^{-1}\left(x_{t-1},\epsilon_{t-1}\right)\\ z_{t-2} & =f_{\theta }^{-1}\left(x_{t-2},\epsilon_{t-2}\right)\\ \; & \vdots \\ z_{1} & =f_{\theta }^{-1}\left(x_{1},\epsilon_{0}\right) \end{array}$$

where $f_{\theta }^{-1}$ is the inverse of $f_{\theta }$.

**Figure 3:**
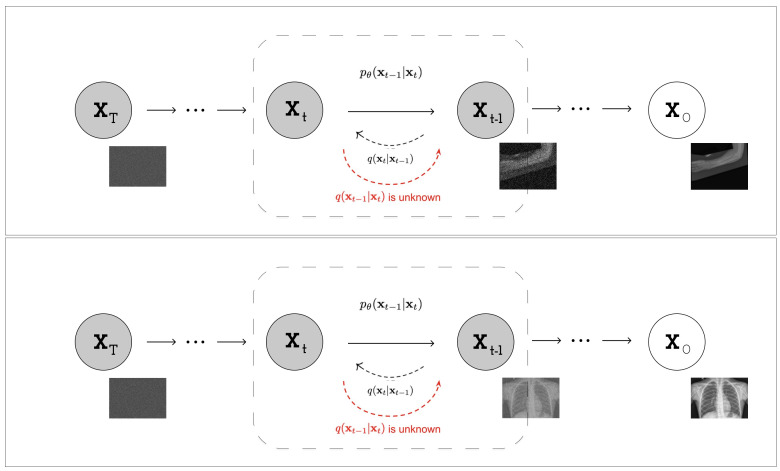
Visual representation of the inverse VDM process: reconstructing a medical image from Gaussian noise by reversing noise addition over time.

The reverse-time generative model (as you can see in Figure 3) can be used to sample from the diffusion process by first sampling the final observation $x_{T}$ from the prior distribution then sampling the noise vectors $\epsilon_{1:T-1}$ and computing the corresponding latent variables $z_{1:T}$ using the reverse-time generative model.

Finally, the diffusion process is run in forward time to obtain the sequence of observations $x_{0:T}$, as described in Section 2.3.

The VDM model is composed of three main components: the ScoreNet, encoder, and decoder. Together, these components form the core of our variational diffusion models (VDMs).

### 2.4. Latent Diffusion: The Encoder/Decoder Models

In order to perform latent diffusion, we first need to map our high-dimensional input data, such as images, to a lower-dimensional latent space. This is performed using an initial encoder. In our algorithm, the initial encoder is implemented using a ResNet architecture.

ResNet is a type of neural network that is designed to facilitate the training of very deep networks. It does this by introducing shortcut connections that allow information to bypass certain layers, which helps prevent the problem of vanishing gradients.

ResNet is used to encode the input images by first passing them through a dense layer and then through a series of residual blocks. The number of residual blocks is determined by the n_layers hyperparameter, which can be adjusted when the encoder is created. The output of ResNet is a tensor that describes a lower-dimensional representation of the input image. This tensor is then passed through another dense layer to produce the output tensor params.

At the end of the diffusion process, we need to map the lower-dimensional latent representation back to a full-sized image. This is performed using a decoder. The decoder is implemented using another small ResNet architecture, which takes the latent representation as input and generates the output image. Additionally, a mean field Bernoulli observational model is used in the decoder to account for the final observed noise in the data.

The small ResNet used in the decoder is similar to the one used in the encoder. It consists of a series of residual blocks, followed by a dense layer, which maps the latent representation to the output image space. The number of residual blocks is determined by the n_layers hyperparameter, which can be adjusted when the decoder is created. The output of ResNet is a tensor that describes the logits of the Bernoulli distribution.

To obtain the output image, the logits tensor is rearranged to match the shape of the input image and passed through a Bernoulli distribution. This Bernoulli distribution accounts for the final observed noise in the data. The independent function is used to ensure that each pixel is modeled independently by the distribution, and the final output is a Bernoulli distribution.

### 2.5. ScoreNet

The ScoreNet is a neural network model used in a diffusion model to remove noise from images. The model takes in a noisy image, a time step, and a conditioning signal as inputs. The architecture of the model consists of a ResNet, where the conditioning signal is used in a FiLM style to condition the ResNet. The conditioning signal is concatenated with the time step and passed through two fully connected layers with the activation function “swish”. The output of these layers is then passed through a final fully connected layer to produce the conditioning signal used in the ResNet. The ResNet takes the noisy image and the conditioning signal as inputs and outputs a cleaned image, obtained by adding the output of the ResNet to the input image. The ResNet architecture used in the model has a number of layers, where each layer consists of a residual block with two fully connected layers and the activation function “swish”. The ScoreNet model provides a powerful tool for denoising images by predicting the noise that is contained in the image and removing it.

The ScoreNet class, implemented as a neural network module, is characterized by an embedding dimension of 128 (denoted as embedding_dim) and 10 layers within a ResNet structure (denoted as n_layers). Within its call method, it first ensures that *t* (derived from input $g_{t}$) is a vector and then creates a time-step embedding, temb, using it. This embedding is concatenated with a conditioning variable and passed through a series of dense layers with swish activations to form a conditional vector, cond. Meanwhile, input *z* is passed through a dense layer, and then through the ResNet block with n_layers, using cond for conditioning. Finally, the transformed *z* is added to the output of the ResNet to produce the final output. This architectural design could be leveraged in various generative models or other scenarios where conditioning on certain variables is essential.

## 3. Results

In this section, we present the results of our Medical VDM model in generating medical images. We evaluated our model on a dataset of 1341 images for the chest X-ray modality. We used a batch size of 1 and trained the model for T to equal 20 K time steps globally.

In our paper, we employed high-performance hardware resources for training our model. The system included a Corsair iCUE 4000X RGB case for efficient cooling, an Intel Core i9-10850K CPU running at 3.6 GHz (up to 5.2 GHz with turbo boost), and 32 GB of Team Group T-Force Delta RGB RAM operating at 3200 MHz. The training process was accelerated with the aid of a Samsung 970 Evo Plus 500 GB M.2 Nvme SSD for fast data access and powered by a Corsair RM850x (Édition 2021) PSU. Additionally, we harnessed the immense computing power of an RTX 3090 24GB GPU, enabling us to achieve state-of-the-art results in our research.

The VDM loss measures the Kullback–Leibler (KL) divergence between two joint distributions, which sets an upper limit on the KL divergence of the image marginals. This concept is discussed in the paper by Sohl-Dickstein et al. [11]. Similar to the approach taken in a variational autoencoder (VAE), the idea is that modeling the larger joint distribution might be simpler than modeling the density directly. In simple diffusion models, the forward process involves fixed additive Gaussian noise. If we take enough steps in the forward process, we should be able to accurately learn the reverse process.

**Figure 4:**
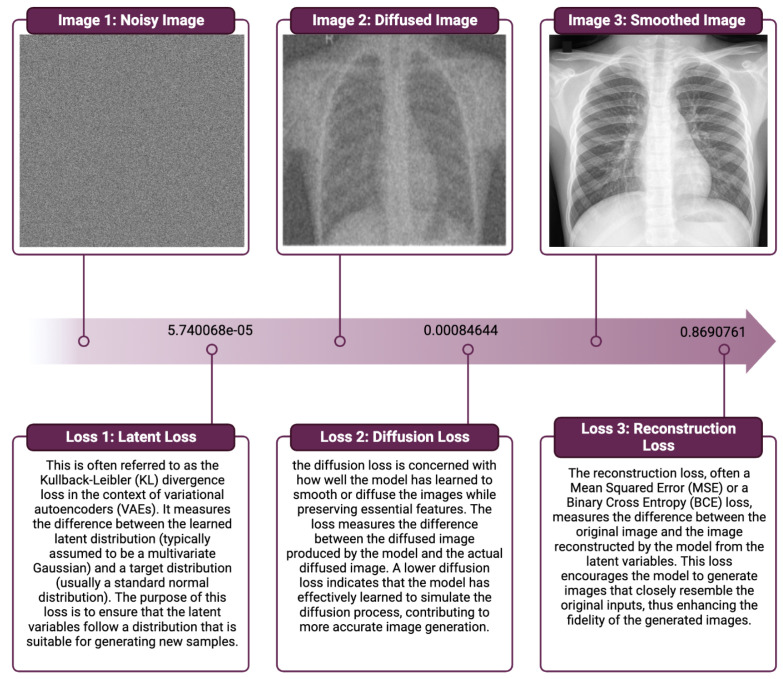
This figure presents the quantitative results of our Medical VDM model on the validation dataset. The various losses, including latent loss, diffusion loss, and reconstruction loss, are depicted. These losses were computed during the training phase to optimize the model. The gradients of these losses with respect to the model’s parameters were used to update the parameters in a manner that minimized the overall loss. The depicted trends provide a comprehensive view of the model’s performance and its optimization process during training.

Figure 4 summarizes the quantitative results of our model on the validation dataset, and these losses are used to optimize the model during training by computing the gradient of the loss with respect to the model’s parameters and updating the parameters to minimize the loss.

From these results, we demonstrate the remarkable performance of our proposed VDM model for medical image generation. The model exhibits a reconstruction loss of 0.869, indicating its exceptional ability to faithfully reproduce the input data. Furthermore, the diffusion loss of 0.0008 and the Kullback–Leibler divergence loss of $5.740\times 10^{-5}$ highlight the model’s capability to efficiently balance the generative process and maintain an accurate representation of the underlying probability distributions.

These outcomes showcase the VDM model’s proficiency in capturing the intricate patterns and structures present in medical images, thereby yielding highly accurate and reliable image generation. The compelling performance metrics substantiate the VDM approach’s potential to revolutionize the medical imaging domain, laying the groundwork for future advancements and applications in healthcare.

**Figure 5:**
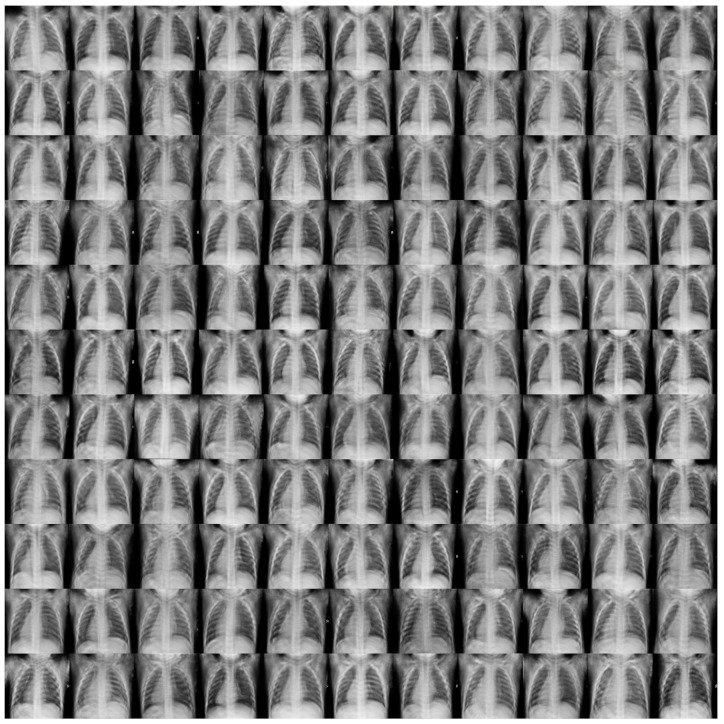
Unbiased, random samples from our chest X-ray generation model, which underwent continuous-time training and used T = 20,000 during image creation. The model’s parameters and hyperparameters are fine-tuned to enhance the quality of the synthesized images, ensuring the generated medical images accurately represent the true anatomical and physiological features.

Figure 5 shows some sample images generated by our model.

We can conclude that our model achieved an average that demonstrates the effectiveness of incorporating clinical knowledge into the model. The generated images also demonstrate the potential for our model to aid medical professionals in accurately diagnosing and treating patients.

In this study, we presented the Medical VDM approach, a method for generating high-quality medical images that accurately reflect the underlying anatomy and physiology. Our experimental results demonstrated the efficacy of the Medical VDM in comparison to current VDM methods. Our results indicate a significant improvement in generating faithful medical images, which can have important implications in various clinical settings.

The Medical VDM has the potential to be used as a valuable tool in medical education and training. By generating high-quality images, it can help teach medical students and healthcare professionals about various anatomical structures and pathological conditions. Furthermore, the enhanced images can be used to simulate complex clinical scenarios, providing hands-on training opportunities and improving diagnostic and treatment skills.

The improved medical image generation offered by the Medical VDM also has the potential to aid clinicians in diagnosis and treatment planning. High-quality images can provide more accurate and detailed information, which can contribute to more precise diagnoses and better-informed treatment decisions. By reducing noise and preserving essential features, the Medical VDM can improve the interpretability of images, particularly in cases where traditional imaging methods struggle.

Ethical considerations and guidelines for the responsible use of generated medical images are paramount. While the Medical VDM approach offers numerous benefits, it is essential to ensure that the generated images are used ethically and responsibly. This includes obtaining informed consent from patients, ensuring data privacy and security, and maintaining transparency about the use of generated images in clinical practice. Furthermore, clinicians and researchers should be aware of the potential biases and limitations of generated images and should continue to rely on their expertise and judgment when making clinical decisions.

In conclusion, our Medical VDM approach represents a significant advancement in the field of medical imaging. By combining the power of VDMs with clinical expertise, the Medical VDM has the potential to enhance medical education, research, and clinical practice, ultimately leading to improved patient outcomes. However, it is essential to address ethical concerns and establish guidelines for the responsible use of generated medical images to ensure that this powerful technology is harnessed in a manner that benefits both patients and healthcare professionals.

## 4. Conclusions and Future Outlook

In conclusion, this study presents an approach to medical image generation, the Medical VDM, which employs variational diffusion models to generate high-quality images while preserving essential features, such as edges. Our experimental findings demonstrate the efficacy of the Medical VDM in producing accurate and reliable medical images, surpassing existing methods. The results showcase the potential of this approach to revolutionize the medical imaging domain and contribute significantly to the advancement of healthcare.

As for the future outlook, several potential research directions can be explored to further improve the performance and applicability of the Medical VDM. One possibility is to investigate the integration of the Medical VDM with other machine learning techniques, such as reinforcement learning, to develop more robust and efficient medical imaging models. Additionally, exploring the application of the Medical VDM in various medical imaging modalities, such as MRI, CT, and ultrasounds, can help broaden its scope and utility in the medical field. It would also be valuable to evaluate the clinical impact of the Medical VDM in real-world scenarios, measuring its effectiveness in aiding diagnosis, treatment planning, and medical education. Finally, as ethical concerns surrounding the use of generated medical images remain crucial, the ongoing assessment and refinement of guidelines for their ethical use should be a priority for researchers and practitioners alike.

## Data Availability

The data presented in this study are available on request from the corresponding author. The data are not publicly available due to licensing agreements.

## Abbreviations

The following abbreviations are used in this manuscript:

VDMs	Variational diffusion models
GAN	Generative adversarial network
VAE	Variational autoencoder
CT	Computed tomography
MRI	Magnetic resonance imaging
FiLM	Feature-wise linear modulation
JAX	Just After eXecution
XLA	Accelerated linear algebra
